# Users’ experiences of an online intervention for bipolar disorder: important lessons for design and evaluation

**DOI:** 10.1136/eb-2017-102754

**Published:** 2017-10-23

**Authors:** Alyson L Dodd, Sara Mallinson, Martin Griffiths, Richard Morriss, Steven H Jones, Fiona Lobban

**Affiliations:** 1 Department of Psychology, Faculty of Health & Life Sciences, Northumbria University, Newcastle-upon-Tyne, UK; 2 Spectrum Centre for Mental Health Research, Division of Health Research, Faculty of Health & Medicine, Lancaster University, Lancaster, UK; 3 Alberta Health Services/ Community Health Sciences, University of Calgary, Calgary, Alberta, Canada; 4 Department of Psychiatry and Applied Psychology, Institute of Mental Health, University of Nottingham, Nottingham, UK

**Keywords:** Mental health, depression, mood disorders, qualitative research, clinical trials

## Abstract

**Background:**

The evidence base for digital interventions for physical and mental health, including severe and enduring mental health difficulties, is increasing. In a feasibility trial, web-based Enhanced Relapse Prevention (ERPonline) for bipolar disorder demonstrated high recruitment and retention rates. Relative to participants in the waitlist control group, those who received ERPonline showed increased monitoring for early warning signs of relapse and had developed more positive illness models.

**Objective:**

To understand users’ motivations and barriers for taking part in an online/telephone-based trial, and for engagement with ERPonline.

**Methods:**

Participants from the trial who had been allocated to receive ERPonline were purposively sampled to participate in telephone-based, in-depth qualitative interviews about their experiences. Interviews (n*=*19) were analysed using framework analysis to identify themes relevant to study aims.

**Findings:**

Participants took part due to the convenient, flexible and rewarding aspects of the trial design, as well as a desire to improve the mental health of themselves and others. Barriers included extensive assessments, practical difficulties and mood. ERPonline was was generally considered to be accessible, relevant and straightforward, but there were individual preferences regarding design, content and who it was for. Several participants reported positive changes, but there was a sense that digital interventions should not replace routine care.

**Conclusions:**

There are a number of barriers and facilitators to consider when evaluating and implementing digital interventions. Individual preferences and human contact were key factors for both trial design and engagement with an online intervention.

**Clinical implications:**

Digital interventions should be co-produced, personalised, interactive and embedded as one component in a broader package of care.

**Trial registration number:**

ISRCTN56908625; Post-results.

## Background

Evidence for feasibility, acceptability and efficacy of web-based mental health interventions is growing rapidly, particularly for conditions like anxiety and depression.^1^ Early evidence indicates that online interventions may have similar utility for more severe and enduring conditions, such as bipolar disorder (BD) and psychosis.^2 3^ Unfortunately, there are insufficient definitive trials to support the scale-up and spread of web-based interventions for people with BD. We know that people with mental health problems may have challenges in engaging with web-based interventions^4^ and that levels of use are likely to be even lower in real world settings than in research settings.^5^ Therefore, it is important to examine users’ subjective experiences of emerging web-based interventions to better understand the factors shaping recruitment, retention and engagement. By identifying and addressing barriers and facilitators to each stage, we can increase the likelihood of people engaging with the programme within and outside trial settings.

For this purpose we built in a nested qualitative study into an online feasibility trial of Enhanced Relapse Prevention (ERPonline) for BD (trial registration number: ISRCTN56908625).^6^ Participants who received ERPonline were invited to interview, as they were able to provide feedback on multiple aspects of the feasibility trial; both user views of the intervention, and the research process. ERPonline is a self-directed web-based intervention to help people develop a coherent working model of their mood changes, recognise and manage triggers and early warning signs (EWS), and develop effective coping strategies (see table 1). Although ERPonline is an unsupported intervention, participants were able to submit technical support queries and potential contributions to a ‘frequently asked questions’ page via the website. The main study was a single blind randomised-controlled trial (RCT). Participants (n*=*96) were randomly allocated to receive access to ERPonline for 12 months, or to a waitlist control (WLC) arm. Both arms received treatment as usual throughout, and the WLCs were given access to ERPonline when the trial ended. Observer-rated assessments were conducted by research assistants via telephone every 12 weeks from baseline to 48 weeks, with additional online self-report questionnaires every 24 weeks. Participants were also contacted by an unblinded member of the team 2 weeks prior to each follow-up assessment being due, to facilitate retention and allow participants to raise any questions or concerns they had about the trial. For further details on the programme content, trial design and findings of the overall study, see Lobban *et al*.^6 7^

**Table 1:** Intervention modules in Enhanced Relapse Prevention (ERPonline)^6 7^

Getting started	How to use the site	Tips for navigation and getting the best from modules, overview of ERPonline (rationale/aims, how to involve a supporter).
	What is bipolar?	Information about bipolar disorder: theories about causes, impact, existing treatments.
Key modules	Mood charting	Online tool to monitor mood on a daily basis in order to identify deviations from usual mood and early signs of a mood episode.
	Life charting	Charting tool for past mood episodes, identifying triggers and developing effective coping strategies for EWS.
	Identifying triggers	Focuses on identifying triggers of previous mood episodes to develop a personalised plan for managing triggers.
Specific moods	EWS (high mood) and coping strategies	Focuses on identifying EWS of high mood to develop a relapse signature for (hypo)mania. Evaluate own strategies currently used to manage (hypo)mania and introduction to helpful coping strategies.
	EWS (low mood) and coping strategies	Focuses on identifying EWS of high mood to develop a relapse signature for depression. Evaluate own strategies currently used to manage depression and introduction to helpful coping strategies.
Wrapping things up	Staying well strategies	Recognising and managing/regulating stress levels and social rhythms, and the impact on interpersonal relationships on mood.
	Staying well plan	Develop personalised plan comprising own staying well strategies and EWS, and helpful coping strategies for mood management.

EWS, early warning signs.

## Objective

To understand the subjective experience of: (1) taking part in an RCT delivered primarily online (2) using ERPonline.

## Methods

### Participants

In the trial, inclusion criteria were (1) research diagnosis of BD 1 or 2 assessed using the Structured Interview for the DSM-IV (SCID);^8^ (2) telephone and internet access, (3) ability to understand written and spoken English, (4) at least three lifetime relapses, one within the previous 2 years, and (5) aged 18 years or over. During online consent for the trial, all participants in the intervention arm (n=46) were invited to take part in a qualitative interview about their experiences. Purposive sampling of those who agreed was used to capture a balance of gender, age and amount of time spent on the ERPonline website. Nineteen people were interviewed.

### Procedure

A member of the research team (MG) conducted all the topic-guided qualitative interviews by telephone. The topic guide^9^ was created by the research team with support from the service user reference group (SURG), who also helped refine the phrasing and flow of questions and prompts through pilot interviews. The topic guide provided structure to the interviews, while allowing some flexibility to manage the flow of the interview and raise new issues where appropriate. Interviews covered the following: participation in research; barriers and facilitators to using ERPonline; and reflections on using online support. Participants were given a £10 shopping voucher as a thank you.

### Data analysis

Interviews were digitally recorded and transcribed verbatim. Transcripts were analysed using indexing and charting methods inspired by framework analysis, chosen for its structured approach to reordering and analysing data as the study was nested in an evaluation of a novel intervention, with specific issues to explore.^9^ Initially, all transcripts were independently read by the interviewer (MG) and a second member of the research team in order to develop a tentative coding frame with thematic headings. The themes were a blend of a priori research interests (related to the direction provided by the topic guide) and thoughts/ideas that had arisen in the interviews. Preliminary codes were tested on a small number of transcripts and were then were checked by SURG to help establish the validity of the teams’ interpretation of the transcript.

After addressing feedback from SURG, the coding frame and themes were agreed and the team began applying it to the transcripts. Data were summarised into individual thematic tables, helping the team to focus on key information and patterns in the data. Where necessary, themes were refined as the analysis proceeded and divergent data were captured.

## Findings

Interviews lasted between 22 min and 88 min. Our findings are structured around the following key themes: the experiences of taking part in the research—in particular the barriers and facilitators to both recruitment and retention; engagement with the ERPonline website; who ERPonline should be aimed at; the likely positive changes that may come from using the site, and the role such an online intervention can have in broader mental health services. These themes are elaborated in detail below.

### Experience of taking part in the research: barriers and facilitators

#### Recruitment

The trial had good recruitment and retention rates and distinctive sample characteristics.^6^ These characteristics (low relapse, more likely to be highly educated and in employment) were mirrored here; 73% (n=14) were educated to degree level or higher, and the same proportion was currently employed. The mean age was 42 years and 53% (n*=*10) were female. Clinical characteristics are given in table 2.

**Table 2:** Participant clinical characteristics

	Mood stabiliser	Past therapy for BD	Current therapy for BD	Total relapse	Diagnosis	ERPonline usage (total visits to website)
1	Yes	No	No	8–20	BD-I	125
2	Yes	No	No	21+	BD-I	5
3	Yes	Yes	No	21+	BD-I	0
4	Yes	Yes	Yes	21+	BD-II	123
5	Yes	No	No	21+	BD-I	3650
6	Yes	Yes	No	1–7	BD-I	1
7	No	No	No	21+	BD-I	29
8	No	No	No	21+	BD-I	2016
9	Yes	Yes	No	21+	BD-I	182
10	Yes	Yes	Yes	21+	BD-I	62
11	Unknown/no	Yes	No	21+	BD-I	9
12	Yes	No	No	8–20	BD-I	85
13	Yes	Yes	No	8–20	BD-II	207
14	Yes	Yes	Yes	8–20	BD-I	96
15	Unknown/no	Yes	No	1–7	BD-I	173
16	Yes	Yes	No	21+	BD-I	515
17	Yes	Yes	No	8–20	BD-I	242
18	Yes	Yes	No	21+	BD-I	13
19	Yes	No	No	8–20	BD-I	129

BD, bipolar disorder; ERPonline, web-based Enhanced Relapse Prevention.

Interview feedback suggests that the diverse modes and sources of recruitment (eg, mental health services, the internet and traditional media) underpinned successful recruitment. In addition, most people found registration and consent straightforward, helping to preserve interest in the programme in the early stages. Intrinsic motivators for participation included curiosity and interest around new ideas and supporting research on BD.

*‘I was intrigued about new research and any new tools that could be available for people with mental health problems especially BD. And my son also has BD.’*(Participant 8).

Alongside being motivated to take part to help understand and manage their mood through accessible support, the most frequently mentioned facilitator was a desire to help other people and be part of a ‘wider community’.

*‘I felt that…this might be a useful tool for me to stay on top of the condition and I just felt motivated to kind of be part of the community of people who are looking to tackle this condition and provide my input.’ *(Participant 6).

#### Retention

Key facilitators for high retention were positive human contact from researchers, and trusting that the intervention and trial protocol had been developed by people with relevant expertise and lived experience.^6^ This human contact would not have featured in a completely online trial using only self-report measures, and should not be underestimated as it may explain higher retention rates compared with online trials without researcher contact.^10^ Facilitators are elaborated in more depth here.

Intrinsic motivators included commitment to continuing the process and a sense of ERPonline potentially being helpful for themselves and others.

*‘I committed to, you know, see it through. And if I’ve committed to seeing something through then unless I’ve got a problem with it, I will do that.’ *(Participant 5).

*‘I hoped that the ERP would really help me, really be a useful tool to help me manage my Bipolar[…] I thought maybe there’d be some new or different things on it that I hadn’t already thought of or knew about.’ *(Participant 18).

Reward was another facilitator. While several participants said they were pleased to have been allocated to the treatment arm, many acknowledged this did not matter as the WLC design ensured they would have access afterwards anyway (notably, retention was higher in the WLC arm in the trial).^6^ Participants also appreciated the shopping vouchers given after follow-up assessments.

*‘…whichever group you were in you still got access to it. So I thought it was definitely worth persevering with…Yeah, that (Amazon vouchers) was definitely a good incentive’* (Participant 17).

While acknowledging vouchers were pleasant, convenience and flexibility appeared to be more important facilitators. Researchers arranged follow-up assessments in the evenings where necessary. User-centred arrangements allowed people to integrate participation into their daily routines with relative ease.

‘*There were no problems as such because the interviews were timed to my, you know, when it was convenient for me[…]having both e-mail and telephone communication was good it meant that I could say when I was free.’ *(Participant 7).

Three people commented that telephone interviews were preferable to face-to-face due to ease of setting up, and feeling more comfortable answering sensitive questions without an interviewer physically present. Many participants felt the interviews were routine due to having a long history of being ‘out’ as bipolar and of divulging personal experiences to research and/or to mental health professionals.

For some, interviews could be seen as helpful:

*‘…I find every time I do an interview like that or a questionnaire it does help me focus which is usually quite helpful and just summarise for myself, right this is how I’ve been the last few months, this is how I am now. And it helps me keep on track…’* (Participant 3).

However, there were comments about extensive, repetitive and time-consuming data collection. Past experience with clinical and research assessments may have made this less of a barrier. This raises the question of whether the trial design suited those who had been more recently diagnosed and had less experience of research.

*‘*… *you get used to sharing your thoughts with people[…]when I was first ill sharing your thoughts with people was quite difficult. But having sort of coped this long I’ve had to share my thoughts with quite a lot of people down the line so it’s more a question of you kind of get used to doing it, yeah?’ *(Participant 9).

One participant reported difficulty remembering mood experiences between follow-ups, and three forgot appointments. Reminder emails and general communication were practical and appreciated.

**‘*…my mood at that time for the last appointment I think was quite high and my concentration was bad. I essentially forgot the appointment […] So it kind of wasn’t me not wanting to do it so much as forgetting…*’* *(Participant 3).

Mood was a barrier to engaging with follow-up assessments. Three participants found assessments challenging or ‘emotionally draining’.

**‘*It was a little uncomfortable at times but that’s because they’re uncomfortable experiences really. So the questions are phrased in a very sensitive way and […] they didn’t make me upset sort of thing […] when you look back you kind of think, oh my God. I was in quite a state at one time.’ *(Participant 7).

While it did not jeopardise their ongoing participation, one participant questioned whether it may prove difficult for people who find it triggering to revisit experiences, or were having significant mood symptoms. As such, it is important to bear in mind the sensitive nature of assessments about mood that can seem routine for some, but triggering for others, in all trials.

### Engagement with the ERPonline site

Activity levels across the whole sample allocated to ERPonline were skewed in the trial.^6^ Those who participated in the interviews were sampled across the range of usage levels (which varied between 0 and 3203 number of page views, median 85 in the main trial).^6^ Participants’ accounts of what determined when and how they used ERPonline illustrated barriers and facilitators to engagement specific to BD and others likely to have relevance to online interventions more generally.

#### Facilitators

Participants commented that they liked how ERPonline looked and its easy-to-navigate modular structure. Most viewed ERPonline as straightforward, normalising and clearly worded.

**‘*It looks like a well-designed site[…]you can come across all sorts of sites that just aren’t user friendly whatsoever[…]it was clear what you had to do and where you had to go. I particularly enjoyed it when it said you’ve completed a module. I don’t know how you feel good when it says you’ve completed a module.’ *(Participant 19).

In line with a systematic review of the acceptability of digital interventions for severe mental health difficulties,^11^ personalised and interactive elements such as videos of people sharing their lived experience, mood and life charting, and relapse prevention strategies were well liked.

**‘*It’s not just kind of like if you were to read a book[…]it’s more than just education and information because you’re actually able to put in your own stuff to personalise it and make it relevant to you.’ *(Participant 15).

In line with this, life and mood charting modules were the most frequently visited during the trial.^6^ These elements elevated ERPonline to having a sense of personal relevance as participants identified with the material and the personal experiences depicted in videos, facilitating self-awareness and feeling less isolated. ERPonline was developed purposely for managing BD, which set it apart from other support.

**‘*…it’s for people with BD. And it gives you far more information about the causes of BD, or possible causes of BD[…] if people haven’t done a WRAP (Wellness Recovery Action Plan) plan, this is something (that is) far more focused on BD that would be probably as helpful to some people that don’t want to do something in a group for example.’ *(Participant 8).

Given this, several participants appreciated ERPonline being there and would recommend it to anyone with BD. A few participants went beyond this to state that ERPonline would be useful for people with recurrent depression, and relatives—supporting the need for web-based support for relatives, for which there is growing evidence.^12^

**‘*I’d recommend it to anybody that’s, not even necessarily got Bipolar, but if they’re just a regular sufferer from a depression. Because I think your bits on the lows are really good as[…]they’re separated aren’t they so people that just suffer the lows would find this really useful[…]I haven’t recommended it to my family yet because they’re (overseas) but, you know, families could easily use this to[…]work through and get a bit of a better idea with what’s going on with their loved one.’ *(Participant 11).

In line with previously reported findings that perceived trustworthiness of the team was important for retention and engagement in both trial arms,^6^ seven participants reported feeling at ease with using the website due to the expertise of the research team. One noted that the information on BD that is available on the internet ranges in its accuracy, so trusted sources are valuable. Another likened the appearance of ERPonline to a ‘government’ medical website, which reassured them.

As would be expected, given they consented to participate in a trial of a web-based intervention, this format was not raised as an issue, as with other online interventions.^13 14^ Participants described feeling like a ‘user’ of the website as an information source or ‘tool’. However, one participant did identify an alliance that made them not want to let people down.

**‘*You did feel like there were real human beings behind it. And so, I think, that did make you feel like you had[…]a relationship and wanting to go back and fill your mood chart in again. You sort of felt like[…]if you didn’t do your mood chart for that day you were sort of letting the chart down in a way.’ *(Participant 16).

#### Barriers

Extrinsic barriers included time constraints and competing demands, such as work, hobbies and social life. This is in line with previous studies exploring user views of web interventions for BD and beyond.^13–15^ Many stated that they felt obliged to use ERPonline due to the research, and that they hadn’t used it as much as they should, which raises questions on engagement in a real world setting. Suggested solutions included reminders, and help to prioritise modules that had not been accessed.

**‘*I haven’t made the decision to use the website or I haven’t been getting reminders from you enough that I should be using the website[…]Too busy, I haven’t clocked on[…]It’s just generally being busy and focusing on other things.’ *(Participant 6).

Access to private space was also an issue, with participants stating they did not want to use ERPonline in public, at work or in front of their family at home. They were concerned about interruption and inadvertently divulging personal information. Privacy is a key concern about digital approaches.^11^

**‘*…when you’re putting in your information about your illness and stuff, I don’t know I feel like that’s personal so I wouldn’t really want to do that in front of other people.’ *(Participant 19).

One participant actively disliked how ERPonline looked, preferring more muted colours.

*‘I just found them too bright and childish.’ *(Participant 6).

Some smartphone apps for supporting severe mental health problems (eg, Actissist) allow customisation of appearance within parameters.^16^ It remains to be seen if this improves usage, but is an important consideration in light of mixed views.

A need for more built-in technical and emotional support with digital interventions was highlighted by participants, with computer literacy raised as a potential issue, and one participant suggesting having a ‘computer therapist on screen’.

A further participant felt the content lacked ‘bite’ and was too superficial, while acknowledging that this may depend on individual knowledge and experience.

**‘*I would have liked to have seen a lot more information at different levels. Sort of the starter and then directing into here is - if you want to get right under the hood of this stuff, then here’s the information.’ *(Participant 4).

This is in line with views on other web-based interventions (eg, Online Bipolar Education Programme) where simplistic and impersonal information was viewed negatively.^13^ There were also mixed views on how interactive and personalised ERPonline was. There is evidence from the wider literature that these elements are valuable, and perceiving added value is important for engagement.^11 15 17^ Efforts must be made to meet different needs by featuring more opportunity for personalisation. This is relevant to mixed views that emerged regarding who should be offered ERPonline and when.

#### Who is ERPonline for?

Some participants believed ERPonline was more useful for those with an established diagnosis. Others stated the opposite and felt that information was pitched at those coming to terms with a new diagnosis, and was overly basic for those with more experience. This may explain quantitative findings that introductory modules, including ‘What is Bipolar?’, were among the least frequently accessed.^6^

**‘*…it was for people for really didn’t know an awful lot. It might be fine for people who have just been newly diagnosed[…]and maybe struggling with, you know, at the early stages of the illness.’ *(Participant 4).

In some ways, mood was a barrier *and* facilitator. Some did not use ERPonline while they were feeling well but felt it offered support when experiencing symptoms.

**‘*I find that when I’m not feeling well that I go back and do it and when I’m feeling well, I’ve got other things I want to do and I can’t be bothered…I haven’t done it for quite a little while because I felt well.’ *(Participant 9).

Others perceived difficulties using the website when unwell, in line with views on other web interventions for both mental and physical health.^5 13 15^ This has implications for retention to online data collection.

While no specific negative impacts were identified, one participant did express concern that mood charting could lead to unhelpful rumination for some people. Any indication of potential harm is important and future research must explore this issue further:

**‘*…mood mapping for me induces rumination. It doesn’t help. And, as I say, it can be difficult to remember certain things and stuff like that. So you’re looking obviously retrospectively and writing things down and that depends on your own mood at the times. So you’re reflecting on something in the past through the eyes of your current mood…and I found that quite destructive actually.’ *(Participant 4).

#### Positive changes

Seven participants reported that they had already been using strategies before ERPonline (eg, mood charting). Eleven reported being more sensitive to triggers and EWS, and better able to proactively and effectively use coping strategies.

**‘*It’s just keeping an eye when I’m a little bit more lively. Making sure I still go to bed at a sensible time. Eating better. Just routine…But most important is the mood monitoring daily…Because without it I won’t know whether I’m going up or down.’ *(Participant 1).

Recognition of their own mood patterns after using ERPonline was specifically mentioned by two participants, one of whom talked about being able to manage medications differently.

**‘*It’s actually made a massive difference to me[…]Well I’ve identified the cycle[…]And that is huge because it means that I can reduce drugs at certain times, increase them at other times[…]and that will benefit my health in the long run because obviously the mood stabilisers and anti-psychotics do have side effects.’ *(Participant 1).

Beyond identification of EWS and implementing coping strategies to avoid relapse, further positive change included having more acceptance over their condition, increased confidence and a renewed sense of control across many domains of functioning. This mirrors findings that ERPonline was related to more monitoring for EWS and positive illness models,^6^ as was the case for online psychoeducation.^10^

**‘*…it (ERPonline) gave me the confidence to actually embark on a relationship for the first time in 5 years. Which is going really, really well. And I think it’s partly that I felt like less of a freak because I knew that there’s a website there and there’s loads of people on the research project. There’s even more people than that got the same symptoms that are described on the website. So that’s been a big help. It’s just given me more confidence…*’** (Participant 2).

This suggests web interventions are able to influence mechanisms of change, even in a basic form, but there is limited quantitative evidence that this has a reciprocal impact on outcomes. However, one participant gave a solid example of how she prevented a relapse because of ERPonline.

**‘*… I was getting a bit high in mood earlier in the month[…]and my husband was quite worried about me. So that’s when I printed off the staying well plan[…]and took it to the doctor and just sort of showed him to sort of explain how I work really… So possibly it helped to prevent a relapse because I was my mood was changing quite a lot[…]and I feel like possibly I prevented - I did prevent a relapse’* (Participant 19).

#### Role of ERPonline in mental health services

Participants typically had a long clinical history. Most had not used an online intervention before, but had used the internet for finding information and forums. The majority felt that ERPonline offered a more tailored, comprehensive and interactive approach by comparison.

**‘*Well it’s just like a complete package really. I mean it wasn’t as thorough as some of the BP (Bipolar) information you can get but as a complete package. I haven’t seen anything like that at all…Well it’s like an A-Z of Bipolar and how you manage the condition. And how you get to know yourself. And the condition in yourself.’ *(Participant 1).

Many people mentioned having had previous psychological therapy and felt that these approaches were helpful. ERPonline was perceived as maintaining and reinforcing usual care, and participants recognised that it could be easily integrated. A key finding reported elsewhere^6^ was that online should not replace face-to-face support; this concern has been raised with other digital interventions.^17^ However, four participants expressed preference for aspects of ERPonline; personal control of the frequency and timing of engagement, and not contending with interpersonal dynamics, waiting lists or large case loads. Three participants specifically said they had shared ERPonline with their psychiatrist or care coordinator. Others were prompted to seek treatment due to using the website, raising an important question about whether service use increases by using online interventions, or whether services are accessed more quickly, reducing further usage down the line:

*‘…seeing it written down made me think, yeah, actually I need to go [to doctor] about this.’ *(Participant 13).

Two people referred to ERPonline as part of a *personalised* ‘toolbox’ of options, resonating with findings that people with BD who had not relapsed for 2 years, and were high functioning, used three or four proactive approaches to staying well.^18^

**‘*Mental health comes as a package which needs to be catered to the individual and the more information you have, the more tools you have in your box, the more likely you are to be able to live a full life’. *(Participant 5).

## Discussion

### Recruitment, retention and engagement

Participants in this study were describing their experiences of taking part in the ERPonline trial and generally spoke favourably about the trial design, which was conducted via telephone and internet.^6 7^ Online trials offer low cost evaluation of interventions, but it is important to consider both intrinsic and extrinsic motivators for participating. For this group, who were generally high functioning and recruited mostly outside of mental health services despite reporting high past usage, these were convenience and flexibility, reward, improving mental health, and a desire to help others and to be part of a community. Human contact as part of the experience was crucial. Barriers were extensive follow-up assessments, forgetting appointments, difficulties remembering past mood and current mood.

Perceived strengths of the ERPonline website design included ease of navigation and use, interactivity, and relevant, comprehensive content. The opportunity to develop individualised staying well plans and mood charts, and specific focus on bipolar, supported the feeling of personal relevance.

Perceived lack of technical and emotional support was a barrier, again highlighting the need for human contact. Appearance and content were not universally liked. Some found the depth of information superficial and lacking novelty, while many participants found it useful and enlightening. There was some discrepancy over whether ERPonline was purely an information source versus an interactive, personalised intervention. This highlights the range of mixed perspectives and preferences.

Opinion also differed on when ERPonline should be offered, to whom and alongside what. Different views and ways of using ERPonline emphasise the importance of personalisation and choice in online interventions to meet diverse needs and preferences. Needs are likely to be different depending on stage of illness. Also, developers should make the targets of the intervention more transparent.

### What did ERPonline change?

ERPonline resulted in more positive models of BD and EWS monitoring,^6^ highlighting that trials should evaluate impact on mechanisms as well as outcomes. These outcomes were elaborated here. Many participants reported that ERPonline brought about a sense of well-being through feeling less isolated, increased confidence, being proactive about engaging in self-management and seeking treatment. Enhanced well-being and self-efficacy, and decreased internalised stigma, are potentially important outcomes for future trials. Participants noted overlap with existing coping strategies and care planning, and potential for integration with routine services, meaning interventions like this may be good for reinforcing existing support.

### Limitations

Participants from the WLC arm were not interviewed, although questionnaire data collected online and reported elsewhere showed similar views regarding online trial designs and the need for web interventions to enhance rather than replace face-to-face services.^6^

The current sample was mostly recruited online, had low relapse throughout,^6^ and none of the participants had dropped out of the trial when they were interviewed, although at least four had used the website very little or not at all (table 2). It is likely that, as in the Bipolar Education Programme trial,^13^ non-adherence will be higher in those recruited from a clinical setting or experiencing more severe mood disruption.

As this was purposeful qualitative research within the context of a feasibility trial, not an exploration of an undefined or unknown experience, framework analysis was used. This can be seen as constraining the data. However, interviews were topic guided, allowing the researchers to explore emerging ideas (which would not be achieved from a survey).

## Clinical implications

These findings are from a feasibility trial of one web-based intervention for BD, yet in line with a systematic review of barriers and facilitators to using mental health interventions online,^4^ this study highlighted several important factors to bear in mind when designing online interventions and the trials to evaluate them.

There were individual preferences in look and feel, and strong indications that online interventions need to be interactive, trustworthy and embedded in a broader social relationship. A diversity of approach is required to give people choice and a range of options to allow them to meet their own needs. Given heterogeneity about what people want and when they want it, and evidence that people can view digital interventions as irrelevant to their individual needs,^17^ digital interventions could offer personalised pathways. Meaningful co-production would help with this, as well as alleviate issues of trust.

It is vital to ensure that web interventions are appropriately resourced. Technical and non-technical support is important to uphold accessibility, motivate people to engage and ensure use is part of a broader support package. This could be done by involving healthcare professionals or engaging peer supporters, as is being done in the online Relatives Education And Coping Toolkit trial.^19^

It is difficult to track and encourage usage of online interventions, where there is no expectation to attend face-to-face sessions. It is unclear if motivation to engage would be present without human contact, and there is already evidence that people are using online interventions for less time in the real world.^5^ It may be that people are more likely to stop using web interventions if expectations are disappointed. The importance of investigating ‘hypothetical acceptability’ prior to use has been highlighted and is an important consideration for future trials.^11^

### Future directions

These findings have broad implications for how to develop, evaluate and implement digital interventions. Future research needs to address several important questions. What is the best way to present information to maximise acceptability and accessibility for a broad range of people? What level of usage is most effective and how can users be supported to engage at that level? How can professionals and other supporters be involved in supporting digital interventions, and where do these fit into healthcare? These questions are important given the growing number of digital interventions for mental health conditions that are currently under development.

## References

[1] Arnberg FK , Linton SJ , Hultcrantz M , et al . Internet-delivered psychological treatments for mood and anxiety disorders: a systematic review of their efficacy, safety, and cost-effectiveness. PLoS One 2014;9:e98118. 10.1371/journal.pone.0098118 24844847 PMC4028301

[2] Hidalgo-Mazzei D , Mateu A , Reinares M , et al . Internet-based psychological interventions for bipolar disorder: review of the present and insights into the future. J Affect Disord 2015;188:1–13. 10.1016/j.jad.2015.08.005 26342885

[3] Naslund JA , Marsch LA , McHugo GJ , et al . Emerging mHealth and eHealth interventions for serious mental illness: a review of the literature. J Ment Health 2015;24:321–32. 10.3109/09638237.2015.1019054 26017625 PMC4924808

[4] Bernard R , Sabariego C , Cieza A . Barriers and facilitation measures related to people with mental disorders when using the web: a systematic review. J Med Internet Res 2016;18:e157. 10.2196/jmir.5442 27282115 PMC4919553

[5] Christensen H , Griffiths KM , Farrer L . Adherence in internet interventions for anxiety and depression. J Med Internet Res 2009;11:e13. 10.2196/jmir.1194 19403466 PMC2762797

[6] Lobban F , Dodd AL , Sawczuk AP , et al . Assessing feasibility and acceptability of web-based enhanced relapse prevention for bipolar disorder (ERPonline): a randomized controlled trial. J Med Internet Res 2017;19:e85. 10.2196/jmir.7008 28341619 PMC5384993

[7] Lobban F , Dodd AL , Dagnan D , et al . Feasibility and acceptability of web-based enhanced relapse prevention for bipolar disorder (ERPonline): trial protocol. Contemp Clin Trials 2015;41:100–9. 10.1016/j.cct.2015.01.004 25602581

[8] First M , Spitzer R , Williams J , et al . Structured clinical interview for the DSM-IV-TR (SCID-I) - research version. New York: Biometrics Research, New York State Psychiatric Institute, 2002.

[9] Ritchie J , Lewis J . Qualitative research practice: a guide for social science students and researchers. London: Sage.

[10] Proudfoot J , Parker G , Manicavasagar V , et al . Effects of adjunctive peer support on perceptions of illness control and understanding in an online psychoeducation program for bipolar disorder: a randomised controlled trial. J Affect Disord 2012;142:98–105. 10.1016/j.jad.2012.04.007 22858215

[11] Berry N , Lobban F , Emsley R , et al . Acceptability of interventions delivered online and through mobile phones for people who experience severe mental health problems: a systematic review. J Med Internet Res 2016;18:e121. 10.2196/jmir.5250 27245693 PMC4908305

[12] Lobban F , Glentworth D , Chapman L , et al . Feasibility of a supported self-management intervention for relatives of people with recent-onset psychosis: REACT study. Br J Psychiatry 2013;203:366–72. 10.1192/bjp.bp.112.113613 24072754

[13] Nicholas J , Proudfoot J , Parker G , et al . The ins and outs of an online bipolar education program: a study of program attrition. J Med Internet Res 2010;12:e57. 10.2196/jmir.1450 21169169 PMC3057316

[14] Poole R , Simpson SA , Smith DJ . Internet-based psychoeducation for bipolar disorder: a qualitative analysis of feasibility, acceptability and impact. BMC Psychiatry 2012;12:139. 10.1186/1471-244X-12-139 22971042 PMC3527357

[15] Donkin L , Glozier N . Motivators and motivations to persist with online psychological interventions: a qualitative study of treatment completers. J Med Internet Res 2012;14:e91. 10.2196/jmir.2100 22743581 PMC3414905

[16] Bucci S , Barrowclough C , Ainsworth J , et al . Using mobile technology to deliver a cognitive behaviour therapy-informed intervention in early psychosis (Actissist): study protocol for a randomised controlled trial. Trials 2015;16:1–10. 10.1186/s13063-015-0943-3 26357943 PMC4566519

[17] Palmier-Claus JE , Rogers A , Ainsworth J , et al . Integrating mobile-phone based assessment for psychosis into people’s everyday lives and clinical care: a qualitative study. BMC Psychiatry 2013;13:34. 10.1186/1471-244X-13-34 23343329 PMC3562160

[18] Russell SJ , Browne JL . Staying well with bipolar disorder. Aust N Z J Psychiatry 2005;39:187–93. 10.1080/j.1440-1614.2005.01542.x 15701069

[19] Lobban F , Robinson H , Appelbe D , et al . Protocol for an online randomised controlled trial to evaluate the clinical and cost-effectiveness of a peer-supported self-management intervention for relatives of people with psychosis or bipolar disorder: Relatives Education And Coping Toolkit (REACT). BMJ Open. In Press. 2017;7:e016965. 10.1136/bmjopen-2017-016965 PMC5541455 28720617

